# Emerging Roles of Mesenchymal Stem/Stromal-Cell-Derived Extracellular Vesicles in Cancer Therapy

**DOI:** 10.3390/pharmaceutics15051453

**Published:** 2023-05-10

**Authors:** Andreas Nicodemou, Soňa Bernátová, Michaela Čeháková, Ľuboš Danišovič

**Affiliations:** 1 Lambda Life a. s., Levocska 3617/3, 851 01 Bratislava, Slovakia; 2 Institute of Medical Biology, Genetics and Clinical Genetics, Faculty of Medicine, Comenius University, Sasinkova 4, 811 08 Bratislava, Slovakia; bernatova52@uniba.sk (S.B.); lubos.danisovic@fmed.uniba.sk (Ľ.D.); 3 Centre for Tissue Engineering and Regenerative Medicine—Translational Research Unit in the Branch of Regenerative Medicine, Faculty of Medicine, Comenius University, Bratislava, Sasinkova 4, 811 08 Bratislava, Slovakia

**Keywords:** mesenchymal cells, extracellular vesicles, cancer therapy, cell-free therapy, drug delivery vehicle

## Abstract

Despite the tremendous efforts of many researchers and clinicians, cancer remains the second leading cause of mortality worldwide. Mesenchymal stem/stromal cells (MSCs) are multipotent cells residing in numerous human tissues and presenting unique biological properties, such as low immunogenicity, powerful immunomodulatory and immunosuppressive capabilities, and, in particular, homing abilities. Therapeutic functions of MSCs are mediated mostly by the paracrine effect of released functional molecules and other variable components, and among them the MSC-derived extracellular vesicles (MSC-EVs) seem to be one of the central mediators of the therapeutic functions of MSCs. MSC-EVs are membrane structures secreted by the MSCs, rich in specific proteins, lipids, and nucleic acids. Amongst these, microRNAs have achieved the most attention currently. Unmodified MSC-EVs can promote or inhibit tumor growth, while modified MSC-EVs are involved in the suppression of cancer progression via the delivery of therapeutic molecules, including miRNAs, specific siRNAs, or suicide RNAs, as well as chemotherapeutic drugs. Here, we present an overview of the characteristics of the MSCs-EVs and describe the current methods for their isolation and analysis, the content of their cargo, and modalities for the modification of MSC-EVs in order for them to be used as drug delivery vehicles. Finally, we describe different roles of MSC-EVs in the tumor microenvironment and summarize current advances of MCS-EVs in cancer research and therapy. MSC-EVs are expected to be a novel and promising cell-free therapeutic drug delivery vehicle for the treatment of cancer.

## 1. Introduction

Mesenchymal stem/stromal cells (MSCs) are multipotent, nonhematopoietic adult somatic stem cells that are present in multiple human tissues, including bone marrow, adipose tissue, amniotic fluid, dental pulp, umbilical cord blood, Wharton’s jelly, etc. [1]. Moreover, MSCs are an important component of the tumor microenvironment (TME) [2]. Due to their unique properties, such as low immunogenicity and powerful immunomodulatory and immunosuppressive capabilities, in particular homing abilities, as well as their contribution to tissue regeneration and repair capabilities, MSCs have become ideal candidates for cell-based therapies, which is further supported by thousands of patients administered with MSCs in clinical trials for the treatment of various diseases, including graft-versus-host disease, hematologic and solid malignancies, bone/cartilage defects, and cardiovascular, autoimmune, or neurologic diseases [3]. Initially, the therapeutic effects of MSCs’ research were attributed to their engrafting and differentiation capacity [4], but current studies indicate that the main therapeutic effects of MSCs are mediated by paracrine mechanisms, through the secretion of a wide array of growth factors, cytokines, and extracellular vesicles (EVs) that collectively contribute to enhancing tissue repair and mitigating inflammatory and immune responses [5,6]. It has been shown that MSCs are involved in tumor development, including tumorigenesis, tumor growth, and metastasis, as well as fine regulation of the TME. However, the exact mechanism of how MSCs affect tumor development and progression is still controversial [7]. MSCs play a double-faced role in tumorigenesis and progression: on one hand, they provide a framework for anchoring tumor cells in the tumor stroma and promote tumor progression through secreting pro-tumorigenic factors [8], supporting tumor angiogenesis, initiating epithelial–mesenchymal transition (EMT), differentiating into tumor-associated fibroblasts [9], and disrupting immune surveillance [10,11,12]. On the other hand, there are many studies demonstrating that MSCs could suppress tumor growth by silencing angiogenesis and increasing inflammatory cell infiltration, apoptosis, and cell cycle arrest, as well as inhibiting the AKT and Wnt signaling pathways [11]. 

To effectively utilize MSCs´ therapeutic potential, it is essential to understand the underlying biological mechanisms and corresponding molecular pathways. Various mediators have been identified that facilitate the crosstalk between MSCs, the tumor microenvironment, and tumor cells; however, there is no doubt that MSC-derived extracellular vesicles (MSC-EVs) play one of the key roles in the interaction between MSCs and tumor cells [13].

Extracellular vesicles (EVs) are a heterogenous group of lipid bilayer membrane organelles of different size (~30–3000 nm), secreted by both prokaryotic and eukaryotic cells [14]. EVs were initially thought to be garbage bags for the elimination of not-needed compounds of the cells [15]. Nevertheless, currently they are considered to be part of the important additional mechanism for intercellular communication, allowing cells to exchange proteins, nucleic acids, and lipids [16]. Moreover, EVs have shown tremendous potential for biomarker discovery in the diagnostics of various diseases, including cancer [17].

MSC-EVs represent almost all properties of maternal cells, in terms of paracrine effects and immunomodulatory functions. In addition, MSC-EVs with defined cargos, such as miRNAs or small-molecule drugs, have been used as a promising strategy for the treatment of different diseases, including cancer. Even more, genetically engineered miRNAs can be used in restoring signaling pathways disrupted in cancer [18]. In this review, information on MSC-EVs’ biogenesis, their contents, and natural functions in the TME is presented, further complemented with an overview of the currently available MSC-EVs’ isolation and characterization technologies intended for therapeutic applications. In addition, we summarize the production and drug loading methods of MSC-EVs and their possible modifications for targeted drug delivery for the treatment of cancer. Finally, the current status of MSC-EV-based therapies for cancer treatment in preclinical and clinical studies is outlined. 

## 2. MSC-Derived Extracellular Vesicles

### 2.1. Classification and Biogenesis

EVs were first mentioned in the late 1960s when Bonucci [19] and Anderson [20] observed small vesicles, about 100 nm in size, secreted by chondrocytes. At the same time, it was reported by Wolf that platelets also release small EVs—at that time mentioned as “platelet dust” and described as a subcellular material originating from platelets in normal plasma and serum that possess significant clogging activity [21]. The MSC-EVs themselves were originally referred by Lai et al. [22] as 50–100 nm particles isolated from MSC-conditioned media and possessing cardioprotective paracrine effects. Since then, several subtypes of MSC-EVs have been identified, including exosomes, ectosomes, microvesicles, membrane vesicles, and apoptotic bodies [23]. In addition, new 35 nm sized EVs were identified by asymmetric-flow field-flow fractionation and termed “exomeres” [24]. Very recently, another distinct EV population, differing in protein and RNA composition and containing high quantities of extracellular RNA termed “supermeres”, was discovered [25]. In addition, the last two mentioned populations were shown not to contain a lipid bilayer, and thus they present as non-vesicular structures with biological activity, termed ”non-vesicular extracellular particles” or “non-vesicular extracellular nanoparticles”, respectively [26].

**Figure 1:**
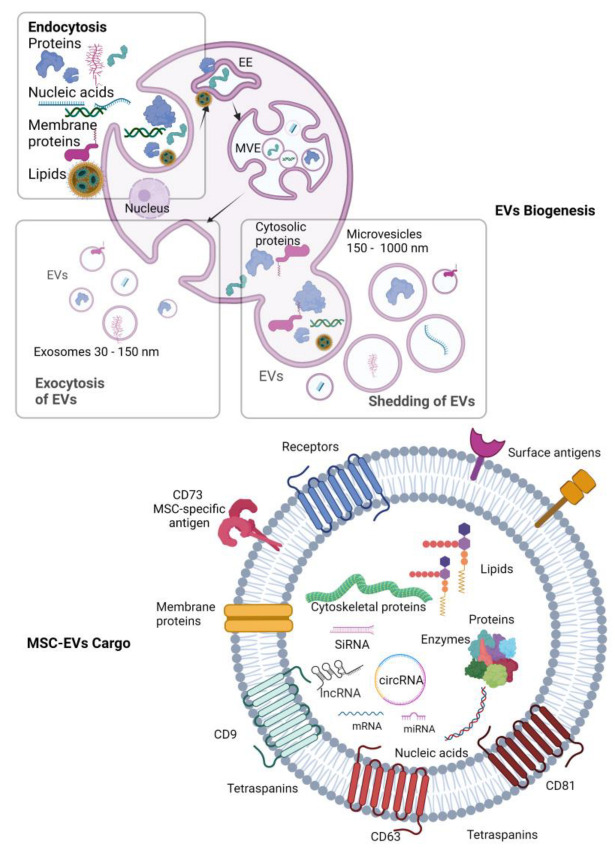
Schematic representation of the biogenesis and cargo of MSC–EVs. Extracellular vesicles (EVs) are a heterogenous group of lipid bilayer membrane organelles of different size (~30–3000 nm). Their subpopulations are derived via distinct pathways. Exosomes which are formed by inward budding of the endolysosomal membrane create early endosomes (EEs) during the maturation of multivesicular endosomes (MVEs) and are released by exocytosis upon fusion of MVEs with cell surface. Microvesicles are formed by shedding out from the cell plasma membrane. MSC-EV cargo comprises luminal-cargo-containing proteins, nucleic acids, peptides, amino acids, and lipid derivates surrounded by a lipid bilayer membrane. MSC-EVs contain transmembrane proteins, lipid-anchored membrane proteins, surface proteins, including MSC-specific proteins (e.g., CD73), soluble proteins, transport proteins (tubulin, actin, and actin-binding molecules), tetraspanins (CD9, CD63, CD81, and CD82), cell adhesion proteins, integrins, ESCRT proteins, enzymes, heat-shock proteins, and various types of RNAs and DNAs6. Created by BioRender.com.

Traditionally, based on their biogenesis, MSC-EVs are divided into two main groups: (1) EVs formed by inward budding of the endolysosomal membrane during the maturation of multivesicular endosomes (MVEs), and released by exocytosis upon the fusion of MVEs with the cell surface [27,28]; and (2) EVs which are formed by shedding out from the cell plasma membrane (Figure 1). The former ones termed as exosomes and the latter ones as microvesicles (microparticles/ectosomes) are the most representative vesicle types of those two groups. The major distinguishing feature between microvesicles and small EVs is their approximate size distribution, ranging from ∼150 to 1000 nm and ∼30 to 150 nm, respectively [29,30]. 

More interestingly, only recently Fordjour et al. presented a new shared, stochastic hypothesis of exosome biogenesis. They showed experimentally that efficient budding from exosomes can occur at the plasma membrane too, and far more efficiently from the plasma membrane than the endosome itself. Their observations also indicate the possibility that most of what we currently know about exosomes has likely come from studies of plasma-membrane-derived vesicles [31]. Additionally, the recent study of Jeppesen et al. has proposed a new model for the active secretion of extracellular DNA through an autophagy- and multivesicular-endosome-dependent but exosome-independent mechanism. They employed the use of high-resolution density gradient fractionation and direct immunoaffinity capture to precisely characterize the RNA, DNA, and protein constituents of exosomes and other non-vesicle material. The membrane-linked protein Annexin A1 was proposed as a unique marker for microvesicles shed directly from the cell membrane. These findings provide a framework for a better understanding of the heterogeneity of EVs and may additionally lead to the proposal that the composition of exosomes should be evaluated [32].

Due to the fact that in the most cases it is impossible to find out the precise biogenic origin of each EV subpopulation, a more comprehensive characterization of the EVs is essential. The general recommendation in the research field, endorsed by the International Society for Extracellular Vesicles (ISEV), is to use “extracellular vesicle” (EV) as the generic term for all particles naturally released from the cell that are delimited by a lipid bilayer and cannot replicate, as indicated in the Minimal Information for Studies of Extracellular Vesicles (MISEV) guidelines. The authors discouraged the use of terms such as “exosome”, “microvesicle”, or “microparticle”, but rather generic terminology including the categorization of EVs by physical characteristics [33]. Furthermore, it should be pointed out, given that no consensus has yet emerged on the specific markers of EV subtypes, that assigning EVs to certain biogenesis pathways remains extremely difficult, and therefore unless authors establish specific, reliable markers of subcellular origin, the usage of operational terms for respective EV subtypes that refer either to physical characteristics, such as size or density, particular biochemical composition (Annexin V-stained EVs, or CD63+/CD81 + EVs), a description of cell growing conditions or origin (MSC-EVs, hypoxic EVs, and large oncosomes (EVs referring to tumor cells)), or apoptotic bodies (large EVs formed in the process of apoptosis) is not adequate, and instead terms such as microvesicle or exosome should be considered [33,34].

### 2.2. Cargo of MSC-EVs

The nature and abundance of EV cargoes are cell-type specific and influenced by the physiological/pathological state of the donor cell, stimuli modulating their production, and mechanisms controlling their biogenesis [35]. MSC-EVs typically comprise luminal-cargo-containing proteins, nucleic acids, peptides, amino acids, and lipid derivates, all surrounded by a lipid bilayer membrane which serves as a transport vehicle and protects the luminal cargo from the rough extracellular environment. Interestingly, the lipid bilayer composition of EVs differs from the lipid composition of the plasma membrane of the cell of origin [36]. Consistent with membranes of dead cells, the lipid bilayer of MSC-EVs exposes negatively charged phosphatidylserine on the outer leaflet in contrast with localization at the inner leaflet of the plasma membrane of viable parental cells, which is also the main reason for the negatively charged surface of EVs [37,38].

MSC-EVs contain a broad array of transmembrane proteins, lipid-anchored membrane proteins (e.g., ectonucleotidases CD39 and CD73 and complement-inhibiting proteins CD55 and CD59), EV surface-associated proteins (e.g., wingless (Wnt) proteins, TGF-β, TNF-α, and FAS ligand), and soluble proteins of the EV lumen [39,40]. Studies have shown that MSC-EVs include large numbers of transport proteins (tubulin, actin, and actin-binding molecules), as well as tetraspanins (CD9, CD63, CD81, and CD82), cell adhesion molecules (CAMs), major histocompatibility complex I (MHC-I) proteins, and integrins, which mediate cellular penetration and invasion and fusion events. In addition, they include signal receptors and immunomodulatory proteins, which are involved in antigen presentation, immune regulation, and the pathophysiology of target cells [41,42]. The incorporation of integrins is of particular importance, as these proteins play a critical role in the organotropism of cancer metastasis and the development of a premetastatic niche [43]. Notably, the markers that are attributed to the surface of MSCs, such as CD29, CD73, CD90, CD44, and CD105, are also found on the surface of MSC-EVs, thus allowing the identification and characterization of MSC-EVs, e.g., by flow cytometry [44]. In regard to luminal proteins, mostly presented are GTPases, proteins involved in EVs’ formation (e.g., Alix, TSG101 or Endosomal Sorting Complex Required for Transport (ESCRT) proteins, and heat-shock proteins (e.g., HSP70 and HSP90, which are involved in antigen presentation and the combination of antigenic peptides with MHC-I molecules)) [16,45]. At present, EVs derived from bone-marrow-derived MSCs (BMMSCs), adipose-tissue-derived MSCs (ATMSCs), or umbilical-cord-derived MSCs (hUCMSCs) have gained the most attention. Therefore, in the study of Wang et al. these three types of MSC-EVs were selected for a comprehensive proteomic analysis. A detailed proteomic analysis revealed 771, 457, and 431 proteins in BMMSC-EVs, ATMSC-EVs, and UCMSC-EVs, respectively, while in terms of biological processes, BMMSC-EV proteins were mainly involved in granulocyte activation and regulation of cell migration, and ATMSC-EV and UCMSC-EV proteins were enriched in the leukocyte activation involved in immune response. In addition, UCMSC-EV proteins were also enriched in the collagen metabolic process. As for molecular functions, ATMSC-EV and UCMSC-EV proteins were both significantly enriched in cell adhesion molecule binding, whereas BMMSC-EV proteins were mostly involved in protein complex binding and integrin binding [46].

MSC-EVs naturally carry DNA cargo, including mitochondrial DNA (mtDNA), double-stranded and single-stranded DNA (ssDNA and dsDNA), and viral DNA, as well as RNA molecules containing messenger RNA (mRNA), circulating RNA (circRNA), transfer RNA (tRNA), mitochondrial RNA (mtRNA), long non-coding RNAs (lncRNAs), and microRNA (miRNA) [47,48], while, e.g., BMMSCs or ATMSCs contain extra distinct sets of miRNAs and tRNAs [49]. Notably, RNA cargo, especially miRNAs, forms critical components of the MSC-EVs’ content. The enrichment of miRNAs in MSC-EVs has been extensively studied. A comparative analysis of MSC-EV miRNAs and miRNAs present in MSCs themselves performed by Liu et al. found that 106 miRNAs in MSCs were not detected in MSC-EVs, indicating that the packaging of miRNAs into EVs is not a random but is a regulated process. Furthermore, the group of Liu et al. constructed the EVmiRNA database to show the miRNA expression profiles of different EVs [50].

In addition to protein and nucleic acids, MSC-EVs contain lipids, especially raft lipids, such as ceramides, sphingolipids, cholesterol, and glycolipid phospholipids [51,52]. Furthermore, EVs contain detergent-resistant domains in lipid membrane–lipid rafts. These rafts are not only enriched in lipids but also contain various proteins, such as flotillin [53]. Unlike protein cargo, EV lipid content is usually conserved and cell-type specific. Lipids complete pivotal roles by EV biogenesis, forming and protecting their structure and homeostasis regulation of their target cells by altering their lipid composition, particularly in the cholesterol and sphingomyelin ratio [39].

It is noteworthy that although much is known about the trafficking of cellular cargo to EVs, the underlying mechanism of cargo selection still remains not clearly understood [54]. It should be stated that in contrast to the previous MISEV2014 guidelines, currently, based on MISEV2018 recommendations, there are no typical EV markers needed to be identified on EVs, but careful discrimination of EVs from non-vesicular contaminants, such as viruses, cytosolic proteins, or protein aggregates, is required [33,55]. Moreover, the previous development of ExoCarta (www.exocarta.org (accessed on 24 April 2023)), a database that lists the cargo identified in exosomes [56], Vesiclepedia (www.microvesicles.org (accessed on 24 April 2023)), a community annotation compendium for EVs [57], and the Extracellular RNA Atlas (https://exrna-atlas.org (accessed on 24 April 2023)), an atlas of cell–cell communication mediated by extracellular RNA [58], has allowed researchers to store and compare their data of identified constituents of EVs and provide a general overview of the molecular composition of EVs.

## 3. Methods for MSC-EV Isolation for Therapeutic Application

Generally, EVs have shown potential in biomarker discovery for efficient diagnostics of various diseases [59]. This is particularly important in tumor diagnostics, since materials typical for cancer cells and carcinogenesis are carried by EVs and distributed in various body fluids, making them attractive as biomarkers for noninvasive diagnosis and prognosis of the stage of cancer directly from liquid biopsies [59,60]. For diagnostic purposes, high-purity isolations are not necessary, but a high yield of EVs is the highest priority. However, for therapeutic purposes (e.g., drug cargo delivery systems and molecular reprograming via miRNA/siRNA or immunotherapy), intact, well-defined, and pure EV isolates are essential, in addition to a reproducible and scalable purification protocol [61]. Currently, there are several commonly used protocols for MSC-EV purification, and the ISEV has proposed detailed guidance for these isolation procedures. However, none of these procedures have achieved absolute purification, meaning complete EV separation from other biological products, or have achieved the combination of high recovery together with high specificity of separation. Each approach has advantages and disadvantages, and combining them may be recommended for the best performance [33]. Different MSC-EV isolation methods are highlighted in Table 1.

The most frequently used isolation protocol is based on the ultracentrifugation (UC) technique, in which an increasing centrifugal force from 300× *g* up to 100,000× *g* is applied first to deplete the medium from larger particles and cell debris in more steps, and finally the EVs are sedimented at 100,000× *g*. In the field of EV isolation, the UC-based techniques are considered to be the gold standard and represent approximately one-half of the isolation methods utilized by researchers [29,62]. While this technique significantly enriches EVs in relatively high yields, it is crude and non-specific, and the EV preparations tend to be contaminated with serum and media particles, lipid droplets, protein aggregates, and cell debris. Moreover, the resulting pellet often cannot be resuspended completely, and high shear forces, causing loss in biological activity, aggregation, and rupture of EVs, represent an important issue. Additionally, the composition of the isolated EVs is highly sensitive to a variety of experimental settings, such as type of rotor or tube, which leads to a low consistency of EV isolates obtained with different ultracentrifuges [63,64,65]. Nevertheless, the UC technique requires little methodological expertise, almost no sample pretreatment, and is widely used in clinical settings [66,67,68,69].

An alternative common technique for MSC-EVs’ isolation is ultrafiltration (UF). As in the case of any other membrane filtration method, UF separates EVs based on their size and the molecular weight cut-off (MWCO) of the utilized membrane filter. MWCO is described as the molecular weight where 90% of the component is rejected by the membrane. For most of the EV preparations the separation range is within 10–100 kDa. EVs larger than the pores are held by the membrane, whereas smaller components are moving through the membrane [61,70,71]. UF has been widely used to isolate EVs from diluted samples, such as urine or cell culture supernatants. UF is often performed as centrifugal UF, which is a relatively simple and easy-to-use technique and offers faster isolation times compared to UC. In addition, it is easily scalable and applicable to clinical conditions [66,72]. However, a major drawback of UF is the undesirable enrichment of protein contaminants, EV losses caused by trapping and clogging of EVs on the membrane filters, and morphological changes of EVs due to the implemented shear force, but those might be diminished through careful regulation of the pressure applied on the membrane [73]. Isolation efficiency can be further improved by sequential filtration (SF)—a technique used for the isolation of EVs by successive steps of filtration. SF is capable of isolating intact high-purity functional EVs from large sample volumes (up to 1 L), which has also been implemented in clinical trials [74,75].

Another size-based ultrafiltration isolation method, which has been increasingly applied in the field, is tangential flow filtration (TFF), also known as cross-flow filtration. In TFF, two streams flow tangentially to a hollow filter membrane, which allows the passing of particles smaller than the MWCO from the feed stream into the permeate stream, while larger molecules, such as EVs, remain in the retentate stream and are recirculated and concentrated [75,76]. TFF is more beneficial compared to conventional filters, as in conventional filters fluid flows directly through membrane, often resulting in clogging of the membrane pores. TFF can be easily scaled and adapted to continuous operation, which together with the short processing times makes it another very valuable isolation technique for the large-scale production of EVs [77]. In comparison to UC, it was demonstrated that the shear stress during TFF does not alter the integrity of EVs, thus offering gentler purification of EVs, but on the other hand TFF provides EVs with lower purity than UC [78,79]. The large amount of protein and lipid impurities often require a further purification step. To achieve a better purity of EVs, some researchers coupled TFF with size-exclusion chromatography (SEC) [80,81]. In SEC, a porous stationary phase (e.g., Sepharose or Sephacryl) is utilized to sort out EVs according to their size, while remaining diluted in the mobile phase. SEC is often used to isolate heterogenous populations of EVs, although the separation of distinct subpopulations remains a challenge due to limited resolution. Furthermore, SEC throughput is noticeably limited by column volume. Nevertheless, coupling TFF to an additional chromatographic step enables more efficient removal of unwanted contaminants and yields a similar amount of EVs as compared to UC, concurrently with preserving EV size, morphology, and protein content. Moreover, both techniques can also be used in GMP-compatible conditions. 

Different from the above-mentioned physical-based isolation methods, methods based on affinity interactions allow high-purity, but often low-yield MSC-EV isolations built on EVs’ interaction with the capture molecules attached to different carriers (e.g., magnetic beads, antibodies, chromatography matrices, and polyethylene glycol (PEG)). Ideally, EV biomarkers for immunoselection are bound on the surface of the EV membrane, lacking soluble counterparts, and are solely expressed or highly concentrated on the surface of EVs. The best examples of such biomarkers are proteins from the tetraspanins family, CD9, CD63, CD81, and CD82, in addition to others such as ALIX, Annexin V, EpCAM, or Rab5 [82,83]. The advantages of affinity-based approaches include fast process, easy operation, and high specificity and selectivity for MSC-EVs of interest, e.g., with engineered surface modification. The drawbacks include costly ligands (antibodies or magnetic beads) and optimizations needed by the elution process (often requires non-physiologic pH or high-ion strength power) to maintain EV integrity and function. However, successful elution of intact EVs was shown in studies with antibodies immobilized inside the monolithic columns [84,85]. PEG (e.g., 10% PEG 6000) precipitation—an alternative method based on affinity interactions—was used by Börger et al. [86] in order to isolate MSC-EVs. The results showed a 14× higher yield compared with the traditional centrifugation protocols. The difference in the weight and concentration of PEG significantly influences the yield of exosomes but cannot affect the size of isolated exosomes. Other studies have shown that different PEG densities influence the contents and characteristics of MSC-EVs, so is important to separate EVs of different densities to find distinct subpopulations [87,88].

## 4. Criteria and Methods for MSC-EV Analysis and Validation in Therapeutic Application

It is crucial to comprehensively characterize isolated MSC-EVs appropriately, primarily in terms of their size, morphology, concentration, presence of EV-enriched or loaded markers, and lack of contaminants. In order for the release of MSC-EV preparations to take place for clinical applications, quality release criteria have to be clearly defined. There are ISEV minimal criteria to appropriately validate EVs preparations. Each preparation should be: (1) defined by quantitative measures of the source EVs (e.g., number of secreting cells, volume of biofluid, and mass of tissue); (2) characterized to the extent possible to determine the abundance of EVs (total particle number and/or protein or lipid content); (3) tested for the presence of components associated with EV subtypes or EVs generically, depending on the specificity one wishes to achieve; (4) tested for the presence of non-vesicular, co-isolated components [33]. 

Additionally, there are further criteria and recommendations for applying EV-based therapeutics in clinical trials, discussed extensively in the ISEV position paper on clinical application by Lener at al. [89]. For instance, in addition to the basic characterization of EVs mentioned above, sterile EV preparations for pharmaceutical use must be tested for the absence of viral and microbiological contaminants and must not contain endotoxins above defined levels. Furthermore, as therapeutic activities cannot be proposed only by molecular profiling, qualified in vitro potency assays are required to predict the intended therapeutic potential of EV preparations, at best in a quantifiable manner. For example, based on the premise that MSC-EVs exert immunosuppressive functions in vivo, T-cell proliferative assays have to be applied to determine immunomodulatory properties ex vivo [89,90]. In addition, certainly the whole preparation of EV therapeutics should demonstrate a standardized production process. In 2017 the EV-TRACK platform was created (https://evtrack.org (accessed on 24 April 2023))—a crowdsourcing knowledgebase allowing researchers to first deposit their isolation and characterization protocols before publication and consequently receive references and recommendations on potential deficiencies in the experimental design [91,92]. 

Currently, there are typically two types of analysis, using different methodologies, performed on EV preparations: (a) physical analysis and (b) biochemical/compositional analysis. The physical analysis gives insight into the size and concentration of MSC-EVs and usually is performed by nanoparticle tracking analysis (NTA), electron microscopy, including transmission electron microscopy (TEM) and scanning electron microscopy (SEM), atomic force microscopy (AFM), dynamic light scattering (DLS), and tunable resistive pulse sensing (tRPS). The biochemical/compositional analysis typically gives information regarding the cargo of the isolated EVs. This is based on immunodetection methods (ELISA, Western blot, and flow cytometry), mass spectrometry (MS) proteomic analysis, and RNA and/or DNA sequencing. Detailed comprehensive reviews of methods for EV analysis can be found elsewhere, e.g., by Doyle and Wang [93], Coumans et al. [94], or Szatanek et al. [95].

## 5. MSC-EVs as Drug Carriers

Specific and targeted delivery of drugs to tumors without harming the surrounding healthy tissues is a hopeful wish of many researchers. Significant advances in the development of smart nanocarriers as drug delivery systems have been achieved in recent decades. In particular, lipid-based nanocarriers are used, and liposomes are the preferred pharmaceutical vehicle for drug delivery, which has led to clinical translations in many applications, such as the delivery of anti-cancer drugs, analgetics, immunomodulators, and anti-fungal or anti-viral drugs. In addition to synthetic nanocarriers, the cell-derived EV-based carrier system has attracted great interest in the last few years. In regenerative medicine and oncology, MSC-EVs are already under clinical assessment as potent vectors for future use intended as “cell-free” therapeutics, due to the following outperforming properties: (1) the tremendous potential to overcome barriers created by the tumor environment; (2) the intrinsic homing ability to target tissues—particularly through strong migrating tropism towards tumor sites; (3) the enhanced ability to cross physical and biological barriers, such as the blood–brain barrier, allowing, e.g., the noninvasive treatment of intracerebral diseases; (4) innate anti-inflammatory and pro-regenerative features; (5) good tolerability in the organism leading to longer circulating times—since they are derived from an organism, they are naturally less immunogenic, and additionally often the presence of the CD47 “do not eat me” marker could prevent EVs from undergoing phagocytosis [55,96,97,98,99]. 

**Figure 2:**
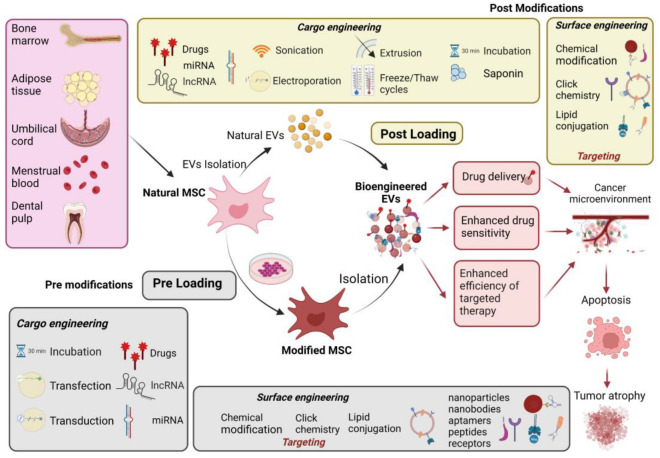
Bioengineering of MSC-EVs and applications of bioengineered MSC-EVs in cancer therapy. Engineered MSC-EVs enhance tumor targeting specificity through targeted drug delivery, increased drug sensitivity of cancer cells, and enhanced efficiency of targeted therapy. Bioengineered MSC-EVs exhibit a higher therapeutic potential, which facilitates the inhibition of cancer progression.6There are two main categories of MSC-EV engineering, cargo engineering and surface engineering, including two main loading strategies: pre-loading and post-loading. Surface modifications are referring to specific targeting of MSC-EVs. Created by BioRender.com.

Although native unmodified MSC-EVs are commonly used in clinical practice, they can be further modified functionally to improve their use as drug carriers (Figure 2). Bioengineered MSC-EVs exhibit higher therapeutic potential since they transfer desired cargo and confer enhanced target specificity. In principle, there are two main strategies regarding how to maximize the therapeutic characteristics of MSC-EVs: cargo engineering (incorporation of therapeutic molecules into EVs) and surface modification engineering (EV mimetics).

### 5.1. Cargo Engineering

Different therapeutic substances, including drugs, proteins, and nucleic acids, can be load into the MSC-EVs. The loading strategies are divided to two main categories:

(1) Pre-loading (parental cell engineering)—before MSC-EV isolation.
(2) Post-loading (direct loading)—after isolation of MSC-EVs.

#### 5.1.1. Pre-Loading

In pre-loading, therapeutic molecules, such as nucleic acids, proteins, and/or small drugs, are loaded into EVs through parental cell engineering (MSCs) during the biogenesis of vesicles, leading to the packaging of the desired molecules into the lumen of newly formed EVs. This approach comprises the loading of functional molecules by increasing their concentration in the cytoplasm of parental MSCs, which can be performed directly by incubating drugs with parental cells (passive loading) or by the genetic manipulation of parental cells via modification of RNA/protein components (active loading).

Transfection and transduction of parental cells via expressing plasmids or retro/lentiviral vectors containing information to create EVs enriched with miRNA precursors, siRNAs, or proteins are currently the most frequently used methods of the active loading strategy [55,80,100]. Lou et al. used plasmid vectors to transfect miRNA miR-122 into ATMSC in order to produce ATMSC-EVs with miR-122 cargo and thereby enhance hepatocellular carcinoma chemosensitivity [101]. The strategy of using expressing plasmids also facilitates the clinical translation of proteins with high molecular weight, e.g., tumor-necrosis-factor-related apoptosis-inducing ligand (TRAIL), a potent anti-cancer molecule, which was transfected to MSCs for the production of TRAIL-enriched MSC-EVs displaying high cancer cell killing efficiency [102]. Nevertheless, the overexpression of a specific protein may cause an imbalance in cell proliferation and leads to apoptosis. This subsequently reduces the proliferation rate of MSCs and the production of EVs. In addition, the pre-loading approach is associated with the risk that EVs are loaded with unwanted proteins or nucleic acids with unpredictable effects on the performed therapy [103].

Passive loading is often performed for the incorporation of small-molecular-weight drugs, such as paclitaxel (PTX) or curcumin. PTX is a hydrophobic mitotic inhibitor with a strong anti-cancer effect. Pascucci et al. incubated MSCs with high dosages of PTX, and the released MSC-EVs containing encapsulated PTX displayed stronger antitumor activity against pancreatic adenocarcinoma compared to PTX alone [104].

#### 5.1.2. Post-Loading

Post-loading is performed after EV isolation. Similarly, as for the pre-loading strategy, exogenous cargoes are loaded passively or actively into EVs. Passive loading is a relatively simple method in which purified EVs are incubated with hydrophobic drugs to allow passive incorporation into the membrane of EVs. The hydrophobic nature of the cargo and the concentration gradient of the molecules determine these methods, which usually exhibit excellent performance for hydrophobic compounds, such as curcumin; however, the stability of passively loaded drugs is still not clear [105,106,107,108].

For hydrophilic molecules such as nucleic acids that cannot incorporate spontaneously into the membrane of EVs, active loading strategies work better in order to temporarily permeabilize the hydrophobic lipid barrier, either physically or chemically, to allow simple penetration of compounds into EVs. The most common approaches to temporarily physically permeabilize the EV membrane are electroporation, sonication, freeze–thaw cycles, and extrusion. These methods were shown to be successful for small molecules as well as macromolecules [109,110,111]. Gomari et al. used electroporation for successful loading of doxorubicin (DOX), one of the most effective antitumor drugs against solid tumors, into the MSC-EVs. DOX-loaded MSC-EVs showed a significant reduction in the murine breast cancer model tumor growth rate [112]. However, some studies have indicated changes in the morphology of EVs and the forming of RNA aggregates, which caused the loss of function of loaded RNAs or destabilized the EVs’ function in vivo; therefore, the potential influence on loaded cargo requires careful consideration [113,114] 

An alternative active post-loading approach utilizes chemicals (transfectants or permeabilizers), such as saponin or triton, to temporarily permeabilize the EV membrane. Saponins are mild surfactants that induce transient membrane destabilization to facilitate the entrance of drug loading into EVs without destroying their lipid bilayer structure. This approach has been shown to be effective mainly for large proteins. Large enzymes over 200 kDa (catalase) have been successfully loaded using saponin detergent [115,116].

### 5.2. Surface Engineering

One of the reasons for the poor therapeutic effect of some chemotherapeutic drugs used in the treatment of carcinomas relates to their systemic and non-targeting effects. Furthermore, the different abilities of distinct types of cells in capturing EVs is another challenge to overcome before EVs will be able to be utilized in clinical practice. Increasing the targeting capacity of MSC-EVs to tumor cells rather than other cells could dramatically improve the efficiency of antitumor therapy. Changing the surface of MSC-EVs, especially the protein composition, can alter the tropism of MSC-EV preparations, thus increasing the local concentration of MSC-EVs at desired sites, improving the therapeutic effects of loaded drug cargo on the target area, and reducing the adverse impact on other areas. Numerous studies have been performed in order to improve the targeting of MSC-EVs using different approaches, which could be classified into three major categories: genetic engineering, chemical modification of target molecule engineering, and hybrid membrane engineering [11,117]. 

Altering the targeting peptide on the surface of MSC-EVs is a highly efficient and direct approach to improving the directing of MSC-EVs. Even if MSC-EVs cross the blood–brain barrier, only systemic administration leads to non-specific accumulation in the lung, liver, spleen, or gastrointestinal track [118]. Therefore, it is necessary to improve the targeting of MSC-EVs directly to the tumor site, e.g., to glioma. Jia et al. coupled the neuropilin-1-targeted peptide to the MSC-EV membrane by click chemistry to improve glioma targeting. Furthermore, they loaded superparamagnetic iron oxide nanoparticles (SPIONSs) and curcumin into MSC-EVs to further enhance the magnetic targeting of MSC-EVs and bioimaging of loaded MSC-EVs. This study demonstrates that such modified MSC-EVs can easily pass to the targeting area, that they are biocompatible, and that they provide appropriate tomography imaging and therapy effects [119]. In another study, Cui et al. coupled the rabies viral protein (RVP) specific for the central nervous system to MSC-EVs to achieve greater cortex and hippocampus targeting [120]. An additional approach is the insertion of glycosyl phosphatidyl inositol (GPI) on the surface of EVs. It functions as an anchoring structure for functional ligands, such as nucleic acids, antibodies, or protein receptors, and subsequently works as a protector of other EV surface proteins against the hydrolytic effect of proteases [121]. Subsequently, the folate receptor can be anchored to the EVs by GPI. Folate is overexpressed on many tumor cells, converse to normal cells where its expression is low; therefore, folate can be used as a targeting ligand for EV delivery [122]. Recently, Feng et al. constructed MSC-EVs named EXO-PH20-FA by introducing folic acid into EVs by genetic engineering, which increased the efficacy of antitumor drug delivery [123].

Utilizing targeting peptides conjugated on the surface of MSC-EVs has been further applied to research on lung cancer, breast cancer, liver cancer, or glioblastoma [117]. Moreover, in combination with metal or gold nanoparticles it is possible to perform imaging (magnetic resonance imaging or computed tomography) to further evaluate the distribution of MSC-EVs in the body in order to better understand pharmacodynamics, targeting, and biodistribution of MSC-EVs. Nevertheless, adding targeting peptides can cause unexpected immunogenicity problems, and the impact of the linking modification is still present. Therefore, it is crucial to well understand the complete attributes of the surface composition, coupling procedures, and the molecular mechanisms of the targeted disease [124,125].

## 6. Distinct Roles of MSC-EVs in Cancer Biology

**Figure 3:**
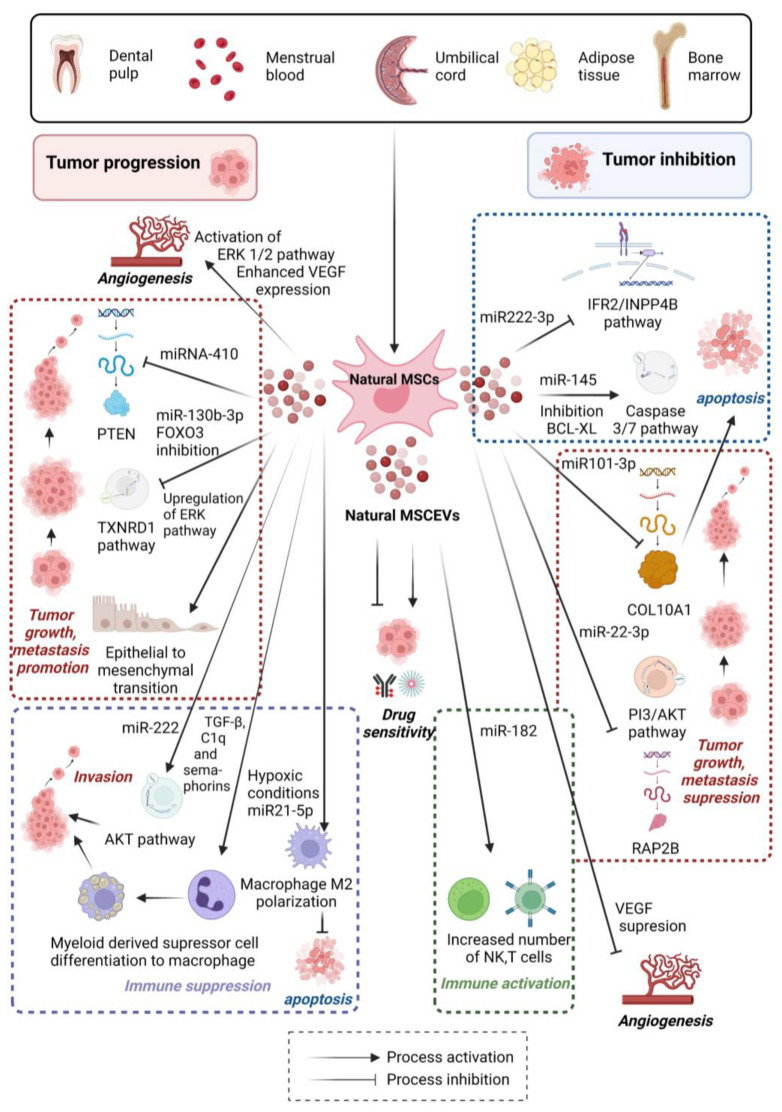
Schematic representation of distinct roles of native MSC-EVs in tumor microenvironment. Mesenchymal stem/stromal cells (MSCs) are present in multiple human tissues, including bone marrow, adipose tissue, amniotic fluid, dental pulp, umbilical cord blood, Wharton´s jelly, etc. Natural MSC-EVs manifest dual role in promoting and inhibiting multiple stages of tumorigenesis. They can mediate tumor proliferation, angiogenesis, apoptosis, tumor invasion (EMT), resistance to drugs, radio- and chemotherapy, and immunosuppression. On the other hand, natural MSC-EVs can exhibit therapeutic effects of modulating immune response, promoting apoptosis of cancer cells, inhibiting EMT and tumor invasion, and enhancing drug sensitivity. Created by BioRender.com.

A number of studies have pointed out the double-edged properties of MSC-EVs in the tumor environment, confirming that MSC-EVs play a dual role in promoting and inhibiting multiple stages of tumorigenesis (Figure 3) [11]. Similarly, as for instance with cancer-cell-derived EVs, MSC-EVs have also been reported to play an important role in mediating tumor proliferation, angiogenesis, apoptosis, tumor invasion, dormancy, or resistance to chemotherapy/radiotherapy [126]. This inconsistency over the dual effect of MSC-EVs on tumorigenesis may be facilitated by many factors, including cell origin, experimental design, culture conditions, method of EV administration, or the tumor microenvironment (TME)–MSC-EV crosstalk. Recently, it seems to be just the crosstalk of MSC-EVs in the TME that is pivotal for cancer progression [127]. Moreover, different physical and chemical factors within the TME, such as hypoxia/anoxia or low/high pH, could strongly affect the behavior of MSCs and thereby alter EVs’ release and content. Depending on the present localization and tumor compartment, MSCs could be heterogeneously activated to receive different chemokine signals for transmitting stimulation or suppression of tumor growth [128]. In addition, controversial findings about the functionality of MSC-EVs may be at least partially attributable to the heterogeneity of the parental MSC populations themselves. MSC-EVs originating from various sources contain different proteins, nucleic acids, and bioactive molecules, both in terms quality and quantity, which can affect not only tumor cells, but also other cells comprising the TME, such as immune cells, tumor-associated macrophages, myeloid-derived suppressor cells, endothelial cells, or cancer-associated fibroblasts [2]. Baglio et al. demonstrated that hBMMSCs and ATMSCs secrete EVs enriched in distinctive miRNA and tRNA species [49]. For instance, in relation to the origin of MSC-EVs, MSC-EVs secreted by hBMMSC-EVs promote the tumor growth and invasion of colorectal cancer or osteosarcoma by the carrying of distinct mi-RNAs or lncRNAs via the upregulation of transforming growth factor-β receptor 3 (TGFBR3) or the elevation of ERB protein expression, respectively [129,130]. In contrast, human-umbilical-cord-derived MSC-EVs (hUCMSC-EVs) carried different types of mi-RNAs, curbing the progression of renal cell carcinoma through T-cell immune response [131]. Analogously, ATMSC-EVs balance the proper differentiation of T helper 17 cells (Th17) and T regulation lymphocytes (Tregs) from naive CD4+ T cell to enhance antitumor ability via miR-10a mi-RNA [132]. A systematic review presented by Christodoulou et al. [133] evaluated that 74% of studies reported a tumor-promotion effect for BMMSCs, 54% for ATMSCs, and only 12% for UCMSCs. These findings suggest that UCMSCs-EVs are the best candidates as drug carriers for further clinical trials. On other hand, almost all studies on tumor-associated MSC-EVs (TAMSC-EVs) reported their strong tumor-promotion effect [2,134]. In the subsections below, the diverse roles of MSC-EVs and their cargo in different tumor environments are discussed, and the respective information (source of MSC-EVs, cargo load, tumor promoting/tumor suppressing effect, and mechanism of function) is summarized in Table 2 at the end of the section. 

### 6.1. Tumor Growth

The discussions about the effects of MSC-EVs in carcinogenesis first appeared when Zhu et al. reported that MSC-EVs could promote tumor growth in vivo, similarly to MSCs. They found that BMMSC-EVs support tumor growth in xenograft models of gastrointestinal cancer; however, BMMSC-EVs did not present the same effect on tumor cells in vivo. BMMSC-EVs enhance the expression of VEGF in tumor cells by activating the ERK1/2 pathway to promote tumor angiogenesis [135]. In general, tumor growth is regulated by a variety of growth factor receptors, such as epithelial growth factor receptor (EGFR), platelet-derived growth factor receptor (PDGFR), or transforming growth factor-β receptor (TGFBR). The activation or phosphorylation of the functional domains of these receptors by respective intracellular kinases initiates pro-growth signals through protein kinase B (PKB/ACT), protein kinase C (PKC), or mitogen-activated protein kinase (MAP/ERK) pathways, leading to tumor cell proliferation. Furthermore, it has been presented that tumor-associated miRNAs enriched in MSC-EVs are strongly associated with promoting or blocking cancer cell proliferation. MiRNAs are short (20–25 nucleotides) single-stranded non-coding RNAs that regulate the post-transcriptional gene expression in target cells by binding to the 3´-UTRs of mRNAs [136]. Many studies have shown that in malignant tumors, such as in breast cancer, pancreatic cancer, osteosarcoma, or colorectal cancer, MSC-EVs can exert pro-carcinogenic effects by regulating different signaling pathways or protein expression through specific miRNAs [129,130,137,138]. Dong et al. has shown that the transfer of miR-410 from human-umbilical-cord-derived-EVs (hUCMSC-EVs) promoted adenocarcinoma cell growth through direct inhibition of PTEN expression [139]. A study conducted by Guo et al. demonstrated that MSC-EVs deliver miR-130b-3p to lung cancer cells to promote cancer cell proliferation, migration, and invasion via blocking the TXNRD1 pathway by FOXO3 inhibition [140]. In addition to a higher level of miRNAs, other factors such as increased levels of cytokines or adhesion molecules in MSC-EVs may also be involved in the promotion of tumor growth [141,142].

In contrary to the studies mentioned above, miRNAs, lncRNAs, and proteins enriched in MSC-EVs can also participate in cancer suppression. For example, miR222-3p, which is highly expressed in BMMSC-EVs, suppresses acute myeloid leukemia (AML) cell proliferation and promotes apoptosis by targeting the IRF2/INPP4B signaling pathway [143]. Similarly, ATMSC-EVs inhibit prostate cancer through the delivery of miR-145 to reduce Bcl-xL activity and promote apoptosis via the activation of the caspase-3/7 pathway [144]. BMMSC-EVS also enable the delivery of miR101-3p and suppress oral cancer progression by targeting COL10A1 [145]. MSC-EVs isolated from different sources of MSCs were shown to participate in both promotion and suppression of tumor growth. This depends on the EVs’ content, such as the composition of respective miRNAs or protein cargo that can vary under different conditions. Accordingly, MSC-EVs could transfer opposite signals in the same tumor type associated with distinct subsets of miRNA or distinct protein levels. Nonetheless, more studies are required to elucidate multiple molecular signaling pathways involved in the regulation of tumor growth. 

### 6.2. Metastasis/EMT

Metastasis is a complex process that involves the spread of tumor cells through the bloodstream or lymph vessels from their original site (primary site) to distant parts of the body and the formation of new tumors (metastatic tumors). This process is a vital feature of malignant cells and causes more than 90% of cancer-related deaths [146]. The induction of EMT is a hallmark of aggressive tumors, and cells that submit to EMT are inclined to disseminate and form colonies distant from the original location. Emerging evidence has indicated the fundamental role of EVs in the EMT mediating metastasis [147,148]. Numerous studies have investigated the role of MSC-EVs in metastasis and EMT promotion, whereas their findings are contributing to both stimulating the formation of metastatic tumors as well as provoking dormant cells [149]. For instance, Zhou et al. reported that hUCMSC-EVs promote tumor progression and metastasis in breast cancer via induction of EMT by upregulation of the ERK pathway [137]. Similarly, Li et al. described MSC-EVs transfected with miR-222 promoting tumor invasion and immunosuppression of colorectal tumor cells via ATF3 binding and mediating the AKT pathway [150]. In contrary, there are studies showing the inhibition of the metastatic potential of tumor cells mediated by the cargo of MSC-EVs. It was demonstrated that hBMMSC-EVs loaded with miR-22-3p were able to suppress colorectal cell proliferation, migration, and metastasis invasion by regulation of the RAP2B and PI3K/AKT pathways [151]. Likewise, hUCMSC-EVs inhibit the proliferation and migration of endometrial cancer cells by transferring miRNA-302a and downregulating the AKT signaling pathway and cyclin D1 [152]. The study of Yao et al. identified the key molecule circ_0030167 derived from BMMSCs-EVs, which inhibits the invasion, migration, proliferation, and stemness of pancreatic cancer cells by sponging miR-338-5p and targeting the Wif1/Wnt8/β-catenin axis [153]. Commonly, MSC-EVs play an important role in the establishment of the premetastatic tumor niche, while miRNAs carried by MSC-EVs can regulate tumor invasiveness through multiple mechanisms leading to both stimulatory and inhibitory effects.

### 6.3. Angiogenesis

Angiogenesis is an essential factor for tumor growth and invasion. This process is distinctly regulated by a delicate equilibrium of pro- and anti-angiogenic factors. MSCs themselves can release a whole collection of growth factors and cytokines, such as vascular endothelial grow factor (VEGF), which may promote neovascularization and thus promote tumor cell proliferation [154]. EVs derived from MSCs have the potential to deliver complex pro- or anti-angiogenic information to endothelial cells that are implicated in the angiogenic signaling. It is especially miRNA cargo that was described by Gong et al. to participate in the induction of angiogenesis via BMMSC-EVs [155]. However, the number of studies investigating the role of MSC-EVs in the angiogenesis of tumorigenic tissues is limited and contradictory. For instance, Rosenberger at al. described that human-menstrual-blood-derived MSC-EVs (hMenMSCs-EVs) could facilitate VEGF suppression and inhibit the growth of oral squamous cell carcinoma [156]. Similarly, hMenMSC-EVs also blocked angiogenesis in prostate cancer by the inhibition of VEGF secretion and NF-κB activity and the induction of generating reactive oxygen species (ROS) [157]. In another study, Pakravan et al. demonstrated that BMMSC-EVs can transfer miR-100 to decrease the expression of VEGF through modulation of the mTOR/HIF-1α pathway, thus inhibiting angiogenesis in breast cancer cells [158]. On the other hand, the previously mentioned study of Zhu et al. reported that BMMSC-EVs are capable of increasing the expression of VEGF in tumor cells by activating the ERK1/2 pathway, thus promoting tumor angiogenesis in xenograft models of gastrointestinal cancer [135]. Collectively, existing evidence indicates that MSC-EVs have an inhibitory effect on tumor angiogenesis, and only a few studies suggest the promoting effect. The dual effects of enhanced angiogenesis of non-tumor tissue in contrast to suppressed angiogenesis of tumor tissues are suggested to be properties of regular MSC-EVs.

### 6.4. Immune Response Regulation

MSC-EVs originating from MSCs exhibited therapeutic effects similar to their parental cells in terms of modulating both innate and adaptive immune response. The dual effect of MSC-EVs in the regulation of the immune system is primarily dependent from the parental cells and the functional state of both parental and target cells [48]. MSC-EVs possess multiple roles in the modulation of responses to T cells, B cells, dendritic cells (DCs), natural-killer cells (NK), and macrophages, as it is extensively summarized elsewhere [159,160]. In general, the most recent studies demonstrated that MSC-EVs, similar to their parent counterparts, mediate immunosuppression rather than immune stimulation [127]. Special attention has to be devoted to TAMSC-EVs, while the majority of studies on TA-MSCs suggest that they support tumor growth. TAMSC-EVs can modify tumor progression by different pathways affecting the whole plethora of TME-residing cells [2]. Yang et al. reported that TAMSC-EVs control cell migration through the miR155/SMARCA4 pathway in teratoid rhabdoid tumors [161]. In relation to immunomodulation, Biswas et al. demonstrated that TAMSC-EVs transfer TGF-β, C1q, and semaphorins, which promotes the differentiation of myeloid-derived suppressor cells into macrophages, thus promoting the progression of breast cancer [142]. Furthermore, in the study of Ren et al. it was demonstrated that BMMSC-EVs grown in hypoxic conditions carry miR21-5p, which is capable of inducing macrophage M2 polarization, leading to the inhibition of apoptosis and the promotion lung cancer development [162]. 

Studies that examined EVs derived from non-malignant MSCs have found rather significant immune stimulation effects; for instance, UCMSC-EVs deliver miR-182, leading to the increase in the proliferation rate of T and NK-T cells and thus suppressing the metastatic potential and growth of renal cell carcinoma [131]. Furthermore, Zhou et al. engineered BMMSC-EVs loaded with galectin-9 siRNA and oxaliplatin (iEXO-OXA), which prompted antitumor immunity through tumor-suppressive macrophage polarization, cytotoxic T lymphocyte recruitment, and Treg downregulation, and achieved significant therapeutic efficacy in cancer treatment [163]. When combined, MSC-EVs primarily mediate immunosuppression rather than the immune stimulation effects, especially in the case of TAMSC-EVs. MSC-EVs may act to achieve immunosuppression as a way of preventing excessive inflammatory response, thus protecting the tissue microenvironment. Nevertheless, bioengineered MSC-EVs loaded with distinct substances could prompt antitumor response.

To summarize, MSC-EVs derived from different sources of MSCs could be naturally loaded by different molecular cargo, which causes different effects on specific tumors. The source of MSC-EVs was demonstrated to be important for the final tumor-promoting or tumor-suppressive effects of MSC-EVs. For instance, hUCMSC-EVs have proven to be one of the most promising choices because of their low tumor-promoting potential [164]. Moreover, their lower immunogenicity in comparison to MSC-EVs from other sources makes them more suitable for use in allogenic therapies [152]. Furthermore, Rocarro et al. showed that BMMSC-EVs isolated from multiple myeloma (MM) patients could support MM tumor growth, followed by elevated dissemination to distant bone marrow niches, by transferring of lower or undetectable levels of miR-15a, while BMMSC-EVs isolated from healthy individuals suppress tumor growth by transferring a usual amount of miR-15a. However, in addition to the different miRNA levels, there were also other factors, such as superior amounts of adhesion molecules and cytokines, which might be involved in tumor-promoting effects [165]. In addition, the contradictory results have indicated a need for further research in the development of standardized production conditions, followed by isolation and purification approaches, as the MSC culture conditions and subsequent isolation and purification techniques may significantly affect the overall features of the derived EVs. Furthermore, the main conclusions in some studies are not sufficiently supported by performed experiments, and detailed data are missing to allow their further reproduction. Therefore, it is crucial to describe all the detailed procedures for the reported parameters (as discussed in Section 4), as these can influence the production and relevant content of analyzed MSC-EVs. Finally, these controversial tumor-promoting and tumor-suppressive features of MSC-EVs might be partially caused by the complexity of the TME and the systemic environment of the host, in addition to the origin of tumor malignances.

**Table 2:** Diverse effects of MSC-EVs in cancer therapy.

Tumor Type	Source of MSC-EVs	EV Cargo	Role	Mechanism	Study Model Mode of Cargo Loading	Ref.
MSC-EVs in promotion of tumorigenesis						
Xenograft of human gastric carcinoma SGC-7901	hBMMSCs	n/a	Tumor growth ↑ Angiogenesis ↑	VEGF↑ Activation of ERK1/2 pathway	In vitro/in vivo Native MSC-EVs	[135]
Gastric cancer	hTAMSCs hBMMSCs	HGF G6PD NF-κB	Proliferation ↑ Metastasis ↑ Angiogenesis ↑	c-Myc-HK2 ↑	In vitro/in vivo Co-culture with gastric cancer cells	[141]
Gastric cancer	hUCMSCs	miR-301b-3p	Multidrug resistance ↑ Proliferation ↑ Migration and invasion ↑	TXNIP ↓	In vitro/in vivo MSC transfected with miR-301b-3p oligonucleotides	[166]
Colorectal cancer	hBMMSCs	miR-424	Proliferation ↑ Migration and invasion ↑	TGF-β receptor 3 ↑	In vitro/in vivo Transfected with plasmid vector	[129]
Colorectal cancer	MSCs (not defined)	miR-222	Proliferation ↑ Migration and invasion ↑ Immune escape	ATF3↓ ATF3 mediates AKT pathway via AKT1 inhibition	In vitro/in vivo Transfected with miRNA using Lipofectamine 2000	[150]
Colorectal cancer	hTAMSCs	miR-30a miR-222	Proliferation ↑ Migration and invasion ↑	MIA3 ↓	In vitro/in vivo Cocultivation	[167]
Pancreatic cancer	hUCMSCs	miR-100-5p	Proliferation ↑ Migration ↑	PANC-1 and BxPC3 cells proliferation ↑	In vitro/in vivo Cocultivation	[138]
Breast cancer	hUCMSCs	n/a	Proliferation ↑ Migration and invasion ↑	EMT via ERK pathway	In vitro Native MSC-EVs	[137]
Breast cancer	hTAMSCs	TGF-β, C1q, and semaphorins	Tumor growth ↑ Metastasis ↑	EMT induction Macrophage M2 polarization MDSC ↓	In vitro Native MSC-EVs	[142]
Lung adenocarcinoma	hUCMSCs	miR-410	Tumor growth ↑ Metastasis ↑	PTEN ↓	In vitro/in vivo Transfected with miRNA using Lipofectamine 2000	[139]
Lung cancer	hUCMSCs	miR-130b-3p	Proliferation ↑ Migration ↑ Apoptosis ↓	FOXO3 ↓ Activation of NFE2L2/TXNRD pathway	In vitro/in vivo Transfected with miRNA using Lipofectamine 2000	[140]
Non-small cell lung cancer	hBMMSCs after hypoxia pre-challenge	miR-21-5p	Tumor growth ↑ Proliferation ↑ Invasion ↑	EMT induction Macrophage M2 polarization	In vitro/in vivo Native MSCs	[162]
Osteosarcoma	hBMMSCs	lncRNA PVT1	Tumor growth ↑ Metastasis ↑	ERG stabilization Sponging miR-183-5p	In vitro/in vivo Native MSC-EVs	[130]
Atypical teratoid rhabdoid tumor	hTAMSC	miR155	Tumor growth ↑ Migration ↑	Mediate SMARCA4 pathway	In vitro/in vivo Transfected with plasmid vector	[161]
MSC-EVs in inhibition of tumorigenesis/therapeutic potential for cancer treatment						
Gastric cancer	hUCMSCs	miR-1228	Tumor growth ↓	MMP-14 ↓	In vitro Genetically engineered	[168]
Colorectal cancer	hUCMSCs	miR-431-5p	Tumor growth ↓	PRDX1 ↓	In vitro/in vivo Transfected with miRNA using Lipofectamine 2000	[169]
Colorectal cancer	hBMMSCs	miR-16-5p	Proliferation ↓ Migration and invasion ↓ Apoptosis ↑	ITGA2 ↓	In vitro/in vivo Cell transfection	[170]
Colorectal cancer	hBMMSCs	miR-22-3p	Proliferation ↓ Migration and invasion ↓ Apoptosis ↑	Mediate RAP2B/PI3K/AKT pathway	In vitro Transfected with plasmids for SW480 cells using Lipofectamine 2000	[151]
Pancreatic cancer	hBMMSCs	KrasG12D siRNA	Tumor growth ↓ Apoptosis ↑	siRNA specific to oncogenic KrasG12D mutation	Clinical trial NC03608631 In vitro/in vivo Clinical testing of GMP iExosomes electroporation with siRNA	[68,99]
Pancreatic cancer	hBMMSCs	Paclitaxel	Proliferation ↓	Anti-cancer drug	In vitro/in vivo Passive loading of MSCs	[104]
Pancreatic cancer	hDPMSCs	Gemcitabine (GCB)	Proliferation ↓	PANC-1 and MiaPaca cell proliferation ↓ Anti-cancer drug	In vitro DPMSC passive loading by GCB	[80]
Pancreatic cancer	hBMMSCs	circ_0030167 molecule	Proliferation ↓ Migration and invasion ↓ Stemness ↓	Sponging miR-338-5p Wif1 ↑ Wnt8/β-catenin pathway ↓	In vitro/in vivo Transfected with miRNA fusing Lipofectamine 2000	[153]
Pancreatic cancer	hBMMSCs	iEXO-OXA (galectin-9 siRNA and oxaliplatin)	Tumor growth ↓ Apoptosis ↑	Tumor-suppressive macrophage M2 polarization CD8+ T Cell ↑ Tregs ↓	In vitro/in vivo Electroporation-loaded galectin-9 siRNA surficially modified with oxaliplatin (OXA)	[163]
Pancreatic cancer	hUCMSCs	miR-128-3p	Proliferation ↓ Migration and invasion ↓	PANC-1 cell proliferation ↓ Galectin-3 ↓	In vitro transfection with plasmid vector	[171]
Hepatocellular carcinoma	hBMMCSs	Norcantharidin	Proliferation ↓ Apoptosis ↑	Anti-cancer drug	In vitro/in vivo Post-loading electroporation	[172]
Oral squamous cell carcinoma	hMSCs	Cabazitaxel TRAIL	Proliferation ↓	Anti-cancer drug Apoptosis induction PI3K/Akt/mTOR phosphorylation ↓	In vitro/in vivo Passive loading of MSCs	[173]
Oral cancer	hBMMSCs	miR101-3p	Proliferation ↓ Migration and invasion ↓ Apoptosis ↑	COL10A1 ↓	In vitro/in vivo Transfected with miRNAs using Lipofectamine 2000	[145]
Hamster buccal pouch carcinoma model of oral squamous cell carcinoma	hMenMSCs	n/a	Tumor growth ↓ Angiogenesis ↓	VEGF ↓	In vitro/in vivo Native MSC-EVs	[156]
Breast cancer	hBMMSC	Doxorubicin	Tumor growth ↓	Anti-cancer drug	In vitro/in vivo Doxorubicin loaded by electroporation	[112]
Breast cancer/prostate tumor/rat glioblastoma	hBMMSCs hUCMSCs hDPMSCs hATMSCs	Loaded mRNA	Tumor growth ↓ Apoptosis ↑	Intracellular conversion of 5-FC to toxic 5-FU through yCD::UPRT enzyme	In vitro/in vivo Transduction by retrovirus vector	[100,174]
Prostate cancer	hATMSCs	miR-145	Proliferation ↓ Apoptosis ↑	Caspase-3/7 pathway ↓ Bcl-xL ↑	In vitro/in vivo Transfected with miRNAs	[144]
Renal cell carcinoma	hUCMSCs	miR-182	Proliferation ↓ Metastasis ↓ T cells ↑ NK-T cells ↑	VEGFA ↓	In vitro/in vivo Native MSC-EVs	[131]
Bladder cancer	hUCMSCs	miR-139-5p	Proliferation ↓ Migration and invasion ↓	PRC1 ↓	In vitro/in vivo Transfected with miRNA using Lipofectamine 2000	[175]
Endometrial cancer	hUCMSCs	miR-302a	Proliferation ↓ Migration and invasion ↓ Apoptosis ↑	cyclin D1 ↓ AKT pathway ↓	In vitro Transduction by lentivirus vector using Lipofectamine 2000	[152]
Lung cancer	hMSCs	TRAIL	Apoptosis ↑	Overcomes TRAIL resistance	In vitro Transfection by expression plasmid	[102]
Lung cancer	hBMMSCs	Let-7i	Proliferation ↓ Metastasis ↓	KDM3A ↓ DCLK1 ↑ FXYD3 ↓	In vitro/in vivo Lentiviral vector pLenti-U6-pgkpuro transduction	[176]
Acute myeloid leukemia	hBMMSCs	miR222-3p	Proliferation ↓ Apoptosis ↑	Inhibition IRF2/INPP4B pathway	In vitro Transfected with miRNAs using Lipofectamine 3000	[143]

hUCMSCs, human-umbilical-cord-derived MSCs; hBMMSCs, human-bone-marrow-derived MSCs; hATMSCs, human-adipose-tissue-derived MSCs; hMenMSCs, human-menstrual-blood-derived MSCs; hTAMSCs, human tumor-associated MSCs; hDPMSCs, human-dental-pulp-derived MSCs; 5-FC, 5-fluorouracil; 5-FC, 5-fluorocytosine; lncRNA, long non-coding RNA; miR, microRNA; TRAIL, tumor-necrosis-factor-related apoptosis-inducing ligand;↑ supportion/increasing of the process/function; ↓ inhibition/decreasing of the process/function.

## 7. Applications of MSC-EVs in Cancer Therapy

EVs are supposed to be promising natural nanovesicles with a huge potential for use in a variety of therapeutic and diagnostic applications. MSCs are one of the most prominent producers of EVs, displaying large expansion capacity compared to other cell sources, which is beneficial for clinically feasible production [177]. It is obvious that MSCs exert their functions via paracrine secretion, while the MSC-EVs are one of the key players. Meanwhile, preclinical data together with data from the performed clinical trials have proven the safety and scalability of MSC-EV preparation processes for clinical applications. MSC-EVs show excellent biocompatibility and exceptional biodistribution properties, including the capability to cross biological barriers, low toxicity and immunogenicity, and strong tumor tropism. Moreover, they could be further artificially modified in order to enhance tumor targeting specificity, efficiency, and safety, and are a promising drug delivery vehicle. Bioengineered MSC-EVs can encapsulate desired therapeutics, such as miRNAs, proteins, or chemotherapeutic drugs. Targeted delivery can improve the efficacy of the drug on the tumor site and reduce the possible strong side effects of the loaded drugs, compared to if applied systemically. One of the reasons for the poor curative effect of some chemotherapeutic drugs relates to their systemic effects. In addition, the use of MSC-EVs as natural drug delivery nanocarriers has several benefits over artificial ones, such as liposomes, as EVs exhibit superior systemic retention, allowing them to exert their function even at distant sites and with decreased immune clearance when administered systemically [55]. Furthermore, the capacity of MSC-EVs in the prolonged release of a precise quantity of drugs increases their therapeutic effectiveness due to the extended circulation of drugs and their accumulation in recipient tumor cells [178]. Moreover, MSC-EVs loaded with therapeutic agents, such as certain mRNAs, miRNAs, regulatory RNAs, proteins, and specific drugs, e.g., paclitaxel [104], doxorubicin/adriamycin [112], gemcitabine [80], cabazitaxel [173], norcantharidin [172], and honokiol [179], demonstrated potent anti-cancer activity; they are delivered to the target site with higher efficiency and maintain a good drug release curve [124]. 

Studies have revealed possible functions of MSC-EVs in oncology, and their target tumor tissue, source of MSC-EVs, cargo load, and mechanism of function are summarized in the second part of Table 2. 

Nevertheless, in contrast to therapeutic applications of MSC-EVs in regenerative medicine, or for the treatment of neurodegenerative diseases, sepsis, graft-versus-host disease (GVHD), or autoimmune diseases, in the field of cancer therapy the situation seems to be more complicated [180]. A number of studies have pointed out the double-edged properties of MSC-EVs in the tumor environment, confirming that MSC-EVs play a dual role in promoting and inhibiting multiple stages of tumorigenesis. The complexity of the TME makes utilizing MSC-EVs for the treatment of cancer much more difficult, for instance in comparison to the field of regenerative medicine for the treatment of tissue injuries. This is further highlighted in the number of studies on human MSC-EVs targeting tumors that reached clinical trials. While there are more than 200 current trials involving MSCs, and more than 1000 if evaluating them together with the completed ones, there are only about 12 registered using MSC-EVs as therapeutics and only 1 trial that focuses on cancer treatment via MSC-EVs (listed in www.clinicaltrials.gov (accessed on 26 March 2023), identifier: NCT03608631). As mentioned previously, this phase I clinical trial involves MSC-EVs loaded with Kras^G12D^ siRNA (iExosomes) in treating patients with pancreatic cancer (metastatic pancreatic adenocarcinoma and pancreatic dual adenocarcinoma) with the Kras^G12D^ mutation. It is a dose-escalation study based on previous preclinical testing employing clinical-grade ATMSC-EVs loaded with Kras^G12D^ siRNAs used to treat pancreatic cancer in animal models, showing a robust increase in overall survival without any clear toxicity [68,99]. The clinical trial currently being performed by Kalluri and coworkers from M.D. Anderson Cancer Center should be completed within this year. Hopefully, it will bring positive results and also promote other groups to push their preclinical studies to the clinical phase. 

## 8. Conclusions and Perspectives

Preclinical data have indicated the efficacy of antitumor therapy based on MSC-EVs. The use of EVs as new cell-free nanocarriers of a vast variety of molecules, including miRNAs, mRNAs, functional proteins, or anti-cancer drugs, linked with the possibility of their modification by methods of bioengineering offers a new paradigm for their application in cancer therapy. MSC-EVs provide the same therapeutic features of their parental cells, however, without safety concerns related to possible tumorigenesis or unwanted gene mutations linked to MSC therapies. In addition, unlike liposomes, they are generally less immunogenic, are well tolerated due to their natural origin, and are much more efficient at entering tumor loci, linked to their strong tumor tropism. Although MSC-EVs have proven antitumor potential, there are also controversial studies about their roles in tumor progression. Further research should distinguish different MSC-EV subpopulations and elucidate their respective roles in cancer development. Correspondingly, there is still a lack of standard methods for the preparation and characterization of MSC-EVs due to their heterogeneity, and this could be further transferred to diverse effects of the therapy. In conclusion, the therapeutic application of MSC-EVs is still in under development, and the precise functional mechanism of their action is often unclear. Therefore, further studies are needed in order to take advantage of the huge hidden potential of MSC-EVs and, with their help, to make cancer history.

## Figures and Tables

**Table 1:** Comparison of most common methods for MSC-EV isolation.

Isolation Method	Isolation Principle	Yield	Purity/Specificity	Advantages	Disadvantages
Ultracentrifugation-based techniques	Density- and sized (shape)-based sequential separations	High/intermediate	Low, higher for more sequential steps	“gold standard”	Need for expensive equipment
				Large sample capacity	Long running time
				Reduced contamination risks and costs (if centrifuge is available)	Low reproducibility—sensitive to experimental settings, damage to EVs, and low RNA yield
Ultrafiltration -based techniques	Ultrafiltration: size	Intermediate	Moderate/high	Simple and fast, no limitation on sample volume, and good portability	Filter plugging—loss of sample and shear stress—deformation of EVs, and low protein yield
	SEC: size	Intermediate	High	Reproducibility, purity, preserves EV structure and biological activity, and eliminates unspecific impurities	Long running time, difficult to scale up, co-isolation of large protein aggregates, and need for special equipment
	TFF: size	Intermediate	Intermediate	Gentle, no clogging in membrane pores, pre preserves EVs structure and biological activity, scalable for therapeutic applications	Moderate purity, protein and lipid impurities, and may require an extra purification step
Affinity-based techniques	Immunoaffinity: interaction with specific EV markers (receptor–ligand)	Low	Very high	High specificity and selectivity, possible to isolate specific subfractions, and easy and fast	High reagent costs (antibodies and magnetic beads), low sample capacity, and tumor heterogeneity hampers immune recognition
	Precipitation: solubility or dispersibility with synthetic polymers or PEG	High	Low	Easy to use, no need for special equipment, and large and scalable capacity	Coprecipitation of non-EV contaminants (proteins and polymeric materials)

## Data Availability

Not applicable.
